# Stigma toward schizophrenia among parents of junior and senior high school students in Japan

**DOI:** 10.1186/1756-0500-4-558

**Published:** 2011-12-22

**Authors:** Hatsumi Yoshii, Yuichiro Watanabe, Hideaki Kitamura, Zhang Nan, Kouhei Akazawa

**Affiliations:** 1 Department of Medical Informatics and Statistics, Niigata University Graduate School of Medicine, 1-754 Asahimachi-dori, Chuo-ku, Niigata, Japan 951-8520; 2 Department of Psychiatry, Niigata University Graduate School of Medical and Dental Sciences, 1-754 Asahimachi-dori, Chuo-ku, Niigata, Japan 951-8520; 3 Health Administration Center, Headquarters for Health Administration, Niigata University, 8050 Ikarashi-ninocho, Nishi-ku, Niigata, Japan 950-2181

## Abstract

**Background:**

Stigma toward schizophrenia is a substantial barrier to accessing care and adhering to treatment. Provisions to combat stigma are important, but in Japan and other developed countries there are few such provisions in place that target parents of adolescents. The attitudes of parents are important to address as first schizophrenic episodes typically occur in adolescence. In overall efforts to develop an education program and provisions against stigma, here we examined the relationship between stigma toward schizophrenia and demographic characteristics of parents of junior and senior high school students in Japan. The specific hypothesis tested was that contact and communication with a person with schizophrenia would be important to reducing stigma. A questionnaire inquiring about respondent characteristics and which included a survey on stigma toward schizophrenia was completed by 2690 parents.

**Results:**

The demographic characteristics significantly associated with the Devaluation- Discrimination Measure were family income, occupation, presence of a neighbor with schizophrenia, and participation in welfare activities for people with mental illness (p < 0.05). The mean ± SD score was 32.74 ± 5.66 out of a maximum of 48 points on the Link Devaluation-Discrimination Measure.

**Conclusions:**

Stigma toward schizophrenia among parents of junior and senior high school students was in fact significantly stronger among members of the general public who had had contact with individuals with schizophrenia. In addition, stigma was associated with family income.

## Background

The stigma attached to the psychosis of schizophrenia is the greatest obstacle to improving the lives of affected individuals and their families. Stigma can lead to a loss of opportunities, decreased self-esteem, discouraging experiences in the workplace or criminal justice system, and disparities in access to health care [1]. It can also drive individuals away from society and incline them towards nonadherence to medication [2]. Moreover, among various factors such as ignorance, denial, lack of motivation, absence of information on early psychosis, and lack of access to appropriate interventions, stigma is associated with psychosis remaining untreated [3].

People see the risks of intervention for schizophrenia as concerning two main issues: drug side-effects and stigma or anxiety surrounding the use of the word 'psychosis' [4]. Many people shun individuals with symptoms of schizophrenia and do not accept them in their communities [5,6]. Therefore, someone with schizophrenia, or who has a family member with the illness, might be less willing to acknowledge the symptoms of the disorder and seek medical care. In this way, stigma becomes a substantial barrier to accessing care and adhering to treatment [1,2].

The purpose of our ongoing research in schizophrenia is ultimately to develop an educational program for early detection and intervention of the disease, and the program naturally must include provisions to counteract stigma. Several sophisticated educational programs to counteract stigma have already been developed to provide basic information on schizophrenia and its associated behaviors to various populations under appropriate conditions [7-9], but there have been no studies of the effectiveness of such programs among the parents of adolescents in Japan. The attitudes of parents are important to address as first schizophrenic episodes typically occur in adolescence. Our recent study suggested that such parents can learn basic information about schizophrenia and better discriminate schizophrenia symptoms by completing a quick (13-minute) web-based educational program [10]. Therefore, in this study, targeting the issue of stigma specifically, we evaluated stigma toward schizophrenia among a large sample of parents of adolescents and examined the association between stigma toward schizophrenia and several demographic characteristics of the respondents. We tested the hypothesis that contact and communication with a person with schizophrenia would be important to reducing stigma [11]. Through investigation of this hypothesis, we aimed to determine which factors, including that of contact and communication with a person with schizophrenia, influence stigma toward schizophrenia among parents of junior and senior high school students in Japan.

## Methods

### Setting and participants

The participants were 2,690 Japanese parents of junior and senior high school students (1,410 and 1,280, respectively) who were extracted from 1,370,000 candidates included in a database administered by a Japanese private company specializing in questionnaire research. Consent from the participants was obtained by the research company and the survey was conducted using an Internet website developed by the company. Stratified random sampling was adopted to prevent bias in the gathered sample, and sex and region were used as the stratifying variables, as previously described [10]. The study sample of 2,690 parents mostly comprised those aged 40-50 years (1,904; 70.8%). The study was approved by the Ethics Committee of the Niigata University School of Medicine.

### Questionnaire

The participants completed a questionnaire that requested information on sociodemographic data, including age, occupation, and education. In addition, to survey stigma, the study questionnaire contained the Link's Devaluation-Discrimination Measure [12] (modified for schizophrenia), which uses a 4-point Likert scale (4, strongly agree; 3, tend to agree; 2, tend to disagree; and 1, strongly disagree). Items 1, 2, 3, 4, 8, and 10 were reverse scored. A higher score on the Devaluation-Discrimination Measure indicates greater stigma.

### Statistical analysis

All analyses were performed using the Statistical Package for Social Science (SPSS) version 16.0. A p value less than 0.05 was considered to indicate statistical significance, and all statistical tests were two-tailed. One-way analysis of variance and Student's t-test were used to examine associations between stigma toward schizophrenia and the demographic characteristics of the respondents. Multiple comparisons were performed with post hoc Bonferroni tests for continuous variables [13]. Cross-tabulation was used to examine associations between occupation and both family income and education.

## Results

### Participant characteristics

Regarding the highest level of educational attainment, 1,063 (39.5%) participants had completed college, 766 (28.5%) had completed high school, and 385 (14.3%) had completed junior high school. Eighty-eight (3.3%) participants reported contact with a close friend or family member who has schizophrenia and 222 (8.3%) reported participation in welfare activities for people with mental illness. The association between occupation and family income is shown in Table 1, and that between occupation and education is shown in Table 2. These findings are discussed in our previous article [10].

**Table 1:** Association between occupation and family income

		Occupation(%)					
	**Agriculture, forestry, and fisheries**	**Production labor services**	**Transportation and communications**	**Sales and marketing**	**Service industry**	**Professional**	**Other**

**Family income (US dollars), (%)**							P < 0.001
< 11,000	9.1	1.0	0.0	0.6	1.8	1.9	1.9
11,000-32,000	18.2	5.7	5.7	8.0	12.6	3.9	13.8
32,000-53,000	0.0	19.2	17.7	19.8	25.0	12.0	20.6
53,000-110,000	63.6	56.3	58.2	51.8	50.3	54.4	53.1
> 110,000	9.1	17.8	18.4	19.8	10.3	27.8	10.6

Total	100	100	100	100	100	100	100

Cross tabulation

**Table 2:** Association between occupation and education

Occupation(%)							
	**Agriculture, forestry, and fisheries**	**Production labor services**	**Transportation and communications**	**Sales and marketing**	**Service industry**	**Professional**	**Other**

**Education(%)**							P < 0.001
Junior high school	0.0	1.0	2.9	0.6	0.9	0.2	0.0
High school	54.5	29.2	30.5	23.5	34.1	17.0	32.5
Vocational school/Junior college	36.4	45.7	39.0	53.3	30.0	49.8	30.0
University	0.0	7.1	3.5	1.4	2.1	5.8	4.4
Graduate school	9.1	17.0	24.1	21.2	32.9	27.2	33.1

Total	100	100	100	100	100	100	100

### Link Devaluation-Discrimination Measure

The mean ± SD Devaluation-Discrimination Measure score was 32.74 ± 5.66, from a possible total score ranging from 12 to 48.

The demographic characteristics significantly associated with the Devaluation-Discrimination Measure were occupation, presence of a neighbor with schizophrenia, family income, and participation in welfare activities for people with mental illness (Student's or Welch's t-test and one-way analysis of variance) (Table 3). Specifically, regarding occupation, participants working in the agriculture, forestry, and fisheries sector had the highest score (mean ± SD: 34.64 ± 3.72), and participants earning less than the equivalent of US$11,000 had the highest score (mean ± SD: 33.71 ± 4.92). The results of Bonferroni's correction revealed a significant difference between those participants categorized under professional and those categorized under production labor services and other (both P < 0.05). The mean score of respondents who reported having a neighbor with schizophrenia was higher than that of individuals who did not. In addition, the mean score of respondents who had taken part in welfare activities for people with mental illness was higher than that of individuals who had not (Table 3).

**Table 3:** Distribution of scores on the Devaluation-Discrimination Measure by demographic characteristics of the parents of adolescents

	Devaluation-Discrimination Measure	
	**Mean ± SD**	p*
**Age(years)**		P = 0.635
30-39	32.47 ± 4.29	
40-49	32.80 ± 4.43	
50-59	32.62 ± 4.55	
60-69	32.47 ± 4.42	
**Occupation**		p = 0.001
Agriculture, forestry, and fisheries	34.64 ± 3.72	
Production labor services	32.22 ± 4.27^a^	
Transportation and communications	32.48 ± 4.43	
Sales and marketing	32.92 ± 4.42	
Service industry	32.74 ± 4.13	
Professional	33.63 ± 4.86^b^	
Other	31.78 ± 3.93	
**Family income, (US dollars)**		p = 0.042
< 11,000	33.71 ± 4.92	
11,000-32,000	32.68 ± 4.71	
32,000-53,000	32.44 ± 4.26	
53,000-110,000	32.66 ± 4.36	
> 110,000	33.20 ± 4.70	
**Proximity to person with schizophrenia**		p = 0.004
Yes	34.39 ± 5.40	
No	32.68 ± 4.40	
**Participation in welfare activities for people with mental illness**		p = 0.010
Yes	33.47 ± 4.79	
No	32.67 ± 4.41	

* Student's or Welch's t-test, one-way analysis of variance.

a P < 0.05, production labor services versus professional

b P < 0.05, professional versus other

## Discussion

Among parents of junior and senior high school students in Japan, the mean score for the Devaluation-Discrimination Measure was 32.74 ± 5.66, which is similar to the score reported by Berge et al. (32.86 ± 6.22) [5].

The results of previous studies of stigma and demographic factors have not been consistent. Mann et al. showed that stigma was not significantly associated with age, race, or religion [14], and likewise the present study found that stigma was not significantly associated with age. However, Papadopoulos et al. found that older participants were more likely to believe that people with mental health problems were more aggressive and less intelligent than individuals without such problems, and that the participants' attitude overall was more likely to be characterized by social restrictiveness [15]. Thus, it would appear that stigma differs with respect to factors such as country of residence and study scale.

Our findings showed higher stigma among individuals employed in the agriculture, forestry, and fisheries sector and those with lower family income, and these two groups have substantial overlap in Japan [16]. The reasons for lower family income are numerous, but also include the fact that those employed in the agriculture, forestry, and fisheries sector have the lowest education level of any industry [17]. In a foreign study, stigma was shown also to be related to social class and education [15,18]. These results might explain the higher stigma among those employed in this sector. The reasons for 3-point difference in Link Devaluation-Discrimination Measure scores seen between the agriculture, forestry, and fisheries sector (34.64 ± 3.72) and 'other' sector which scored the lowest (31.78 ± 3.93) in this study, are not entirely clear. However, the latter score concurs with that of the Japanese general public (31.95 ± 5.74) reported by Shimotsu et al. [19]. Greater stigma is likely attributable to differences in knowledge base, attitude, and behavior that arise from lower family income and lower educational attainment.

Stigma toward schizophrenia may also be closely related to contact with people who have the disorder. In this study, we found that individuals who participated in welfare activities for people with mental illness and those with a neighbour with schizophrenia had greater stigma toward schizophrenia (Table 1). This result did not agree with our hypothesis that having contact and communication with a person with schizophrenia would be important to reducing stigma. Our results could be explained by the following findings, however. Penn et al. [20] maintained that "interpersonal factors, such as overall social skill, negative symptoms, and perceived strangeness, may contribute to stigma". Behavior that is menacing, or simply exaggerated or incomprehensible, may produce aversion and fear. The findings of these studies are likely due to the nature of participant contact with schizophrenics. A study in a medical school in Turkey compared attitude changes in students participating in a 3-week psychiatric training program with those participating in an ophthalmology shift and reported no significant difference. The authors suggested that contact during job training might not affect attitudes because of the characteristics of the interaction [21]. Thus, the nature of contact with people with schizophrenia is likely to determine the extent of any stigma toward them. In addition, the disease view engenders a less favorable estimation of the mentally disordered [22]. Therefore, labeling behaviors 'schizophrenic' has a more pessimistic outlook [23]. In light of this, do any provisions created to counteract stigma require a sufficient base knowledge of schizophrenia? A Hong Kong study found that as 'knowledge' based on the mental illness perspective increased, attitudes became more negative [24]. Therefore, unless a sufficient base knowledge is provided to participants in welfare activities for people with mental illness in the form of an educational program, they may become more negative in their attitude as higher knowledge.

## Conclusions

In summary, stigma toward schizophrenia was stronger among members of the general public who had had contact with people with the disorder, as well as those with lower family income. These results contribute to our ultimate research aim of developing an educational program that can combat stigma toward the disease in Japan.

## Competing interests

The authors declare that they have no competing interests.

## Authors' contributions

HY and KA designed the study and drafted the manuscript. YW, HK, and ZN made significant contributions to the content of the paper and were responsible for the final editorial revision. All authors read and approved the final manuscript.

## References

[B1] Esterberg ML Compton MT McGee R Shim R Hochman K Knowledge about schizophrenia and social distance toward individuals with schizophrenia: A survey among predominantly low-income, urban, African American community members J Psychiatr Pract 2008 14 86 93 10.1097/01.pra.0000314315.94791.80 18360194

[B2] Lysaker PH Davis LW Warman DM Strasburger Amy Beattie N Stigma, social function and symptoms in schizophrenia and schizoaffective disorder: Associations across 6 months Psychiatry Res 2007 149 89 95 10.1016/j.psychres.2006.03.007 17156853

[B3] Chong SA Lee C Bird L Verma S A risk reduction approach for schizophrenia: The early psychosis intervention programme Ann Acad Med Singapore 2004 33 630 5 15531960

[B4] De Koning MB N Bloemen OJ van Amelsvoort TAMJ Becker HE Nieman DH van der Gaag Mvan Linszen DH Early intervention in patients at ultra high risk of psychosis: benefits and risks Acta psychiatrica scandinavica 2009 119 426 42 10.1111/j.1600-0447.2009.01372.x 19392813

[B5] Berge M Ranney M Self-esteem and stigma among persons with schizophrenia: Implications for mental health Care Manag J 2005 6 139 144 10.1891/cmaj.6.3.139 16642688

[B6] Corrigan PW Green A Lundin R Kubiak MA Penn DL Familiarity with and social distance from people who have serious mental illness Psychiatr Serv 2001 52 953 8 10.1176/appi.ps.52.7.953 11433114

[B7] Stuart H Reaching Out to High School Youth: The effectiveness of a video-based antistigma program Can I Psychiatry 2006 51 647 53 10.1177/070674370605101004 17052032

[B8] Kadri N Sartorius N The global fight against the stigma of schizophrenia PloS Med 2005 2 0597 9 10.1371/journal.pmed.0020136 PMC1181868 16033301

[B9] Gaebel W Zaske H Baumann AE Klosterkotter J Maier W Decker P Moller HJ Evaluation of the German WPA "Program against stigma and discrimination because of schizophrenia-Open the doors": Results from representative telephone surveys before and after three years of antistigma interventions Schizophr Res 2008 98 184 93 17961985 10.1016/j.schres.2007.09.013

[B10] Yoshii H Watanabe Y Kitamura H Chen J Akazawa K Effect of an education program on improving knowledge of schizophrenia among parents of junior and senior high school students in Japan BMC Public Health 2011 11 1 323 10.1186/1471-2458-11-323 21575259 PMC3123206

[B11] Tanaka G Inadomi H Kikuchi Y Ohta Y Evaluating stigma against mental disorder and related factors Psychiatry Clin Neurosci 2004 58 558 66 10.1111/j.1440-1819.2004.01300.x 15482589

[B12] Link BG Understanding labelling effects in the area of mental disorders: an assessment of the effects of expectations of rejection Am Sociol Rev 1987 52 96 112 10.2307/2095395

[B13] Glantz SA Primer of biostatistics sixth edition 2005 New York: The McGraw-Hill Companies

[B14] Mann CE Factors associated with stigmatization of persons with mental illness Psychiatric Services 2004 55 185 187 10.1176/appi.ps.55.2.185 14762246

[B15] Papadopoulos C Leavey G Vincent C Factors influencing stigma: A comparison of Greek-cypriot and English attitudes towards mental illness in north London Soc Psychiatry Psychiatr Epidemiol 2002 37 430 434 10.1007/s00127-002-0560-9 12242619

[B16] Ministry of Education, Culture, Sports, Science and Technology http://www.nta.go.jp/kohyo/tokei/kokuzeicho/minkan2009/pdf/001.pdf

[B17] Ministry of Education, Culture, Sports, Science and Technology http://www.mext.go.jp/b_menu/hakusho/html/hpad197501/hpad197501_3_106.html

[B18] Dickerson FB Sommerville J Origoni AE Ringel NB Parente F Experiences of stigma among outpatients with schizophrenia Schizophrenia Bulletin 2002 28 143 155 12047014 10.1093/oxfordjournals.schbul.a006917

[B19] Shimotsu S Sakamoto S Horikawa N Sakano Y Reliability and validity of the Japanese version of Link's Devaluation-Discrimination Scale Seishinka Chiryogaku 2006 21 521 528

[B20] Penn DL Kohlmaier JR Corrigan PW Interpersonal factors contributing to the stigma of schizophrenia: social skills, perceived attractiveness, and symptoms Schizophr Res 2000 45 37 45 10.1016/S0920-9964(99)00213-3 10978871

[B21] Arkar H Eker D Influence of a 3-week psychiatric training programme on attitudes toward mental illness in medical students Soc Psychiatry Psychiatr Epidemiol 1997 32 171 6 9130870 10.1007/BF00794617

[B22] Mehta S Farina A Is being 'sick' really better? Effect of the disease view of mental disorder on stigma J Soc Clin Psychol 1997 16 405 419 10.1521/jscp.1997.16.4.405

[B23] Cormack S Furnham A Psychiatric labelling, sex role stereotypes and beliefs about the mentally ill Int J Soc Psychiatry 1998 44 235 247 10.1177/002076409804400401 10459508

[B24] Chou K Mak K Attitudes to mental patients among Hong Kong Chinese: a trend study over two years Int J Soc Psychiatry 1998 44 215 224 10.1177/002076409804400307

